# The effect of Shenmai injection on the proliferation of Rat airway smooth muscle cells in asthma and underlying mechanism

**DOI:** 10.1186/1472-6882-13-221

**Published:** 2013-09-08

**Authors:** Limin Zhao, Jizhen Wu, Xiaoyu Zhang, Hongyan Kuang, Yali Guo, Lijun Ma

**Affiliations:** 1 Department of Respiratory Medicine and Intensive Care Union, Henan Provincial Peoples' Hospital of Zhengzhou University, Zhengzhou 450003, China

**Keywords:** Shenmai injection (SMI), Transient receptor potential vanilloid 1 (TRPV1), Airway smooth muscle cells (ASMC), Intracellular calcium, Airway remodeling

## Abstract

**Background:**

Over-proliferation of airway smooth muscle cell (ASMC) is one of the important contributors to airway remodeling in asthma. The aim of this study was to investigate the effect of Shenmai injection (SMI) on the proliferation of the rat ASMC in asthma.

**Methods:**

Rats were randomly divided into three groups: the control group, the asthma group, and the SMI treatment group. Reverse transcription-polymerase chain reaction (RT-PCR) and immunocytochemistry staining were used to detect the mRNA and protein expression of transient receptor potential vanilloid 1 (TRPV1) and proliferating cell nuclear antigen (PCNA) in rat ASMC respectively. Intracellular Ca^2+^ concentration ( [Ca^2+^]i ) in rat ASMC were measured with Fluo-3/AM by confocal microscopy. The proliferation was detected by MTT assay.

**Results:**

Compared with the control group, the asthma group showed an increased expression of TRPV1 and [Ca^2+^]i in rat ASMC. The expression of PCNA and absorbance of MTT assay in asthma rat ASMC was also significantly increased. SMI could significantly decrease the expression of TRPV1 channel and [Ca^2+^]i in the asthmatic rat ASMC. Furthermore, the expression of PCNA and absorbance of MTT assay in asthmatic rat ASMC was significantly reduced after SMI treatment.

**Conclusions:**

SMI may prevent asthma-induced ASMC over-proliferation probably by inhibiting the expression of TRPV1 channel, which regulates the intracellular calcium concentration.

## Background

Bronchial asthma is a highly prevalent chronic respiratory disease that is seriously hazardous to human health. Because the pathogenesis of asthma has not been clearly identified, only symptom control can be achieved with the current treatment. Therefore, further clarification of the pathogenesis of asthma and searching for more effective treatments are the hot topics of research at present. Airway remodeling is an important pathological feature of asthma and is the pathological basis of irreversible airflow obstruction [1-3]. An increase of airway smooth muscle (ASM) mass due to proliferation and hypertrophy of ASM cells (ASMC) plays an important role in the pathophysiology of airway hyper-responsiveness and remodeling in asthma [4,5].

Calcium is an important second messenger in ASMC. Intracellular Ca^2+^ has been shown to regulate cell proliferation, differentiation, signal transduction, and so on [6-8]. The transient receptor potential vanilloid receptor (TRPV) is an important Ca^2+^ signaling pathway, especially for TRPV1 channel [9]. It has been reported that the loss of function of TRPV1 genetic variant is associated with lower risk in active childhood asthma, with the reason of intracellular Ca^2+^ dysregulation induced by TRPV1 channel [10].

Shenai injection (SMI) is extracted from red ginseng and ophiopogon root. According to traditional Chinese medicine theory, SMI benefits qi, prevents exhaustion, nourishes yin, and replenishes bodily fluids with the lower adverse drug reactions occurrence [11]. SMI is widely used for clinical treatment of qi-yin deficiency in coronary heart disease, chronic pulmonary heart disease, viral myocarditis, heart and respiratory failure, cerebral infarction and malignant diseases [12]. Our previous studies had found that SMI was demonstrated, in a dose-dependent manner, to inhibit the extracellular signal regulated kinase (ERK) transduction pathway and the proliferation of human ASMC, preventing airway remodeling to occur [13]. SMI could significantly down-regulate the activity of Ca^2+^ channel protein in asthmatic rat models and could possibly exhibit a preventive and therapeutic effect on asthma [14]. SMI showed a definite effect on the Fas and FasL protein expression and inhibited diaphragmatic muscle cell apoptosis, explaining its therapeutic effect on diaphragmatic fatigue caused by hypoxia [15]. However, there had no report on the effect of SMI on ASMC proliferation in asthma and its underlying mechanism. Therefore, this study was designed to investigate the effects of SMI on ASMC proliferation, the expression and activity of TRPV1 channel in asthma, with an objective to understand how SMI affects TRPV1 channel-induced ASMC over-proliferation in asthma.

## Methods

### Establishment of rats chronic asthmatic model and grouping

30 male Sprague–Dawley rats (6 weeks old, 180-200 g body weight) were randomly divided into 3 groups: the control group, the asthmatic group, and the SMI treatment group. Each group consisted of 10 rats. The rats were kept in SPF animal research facilities under standard laboratory conditions and received food and water ad libitum. All experimental procedures were carried out in accordance with the NIH Guidelines for the Care and Use of Laboratory Animals and were approved by Chinese Institute of Oriental Medicine Institutional Animal Care and Use Committee. The animals were cared for in accordance with the National Animal Welfare and Protection Law of China.

The modified protocols for rats sensitization and challenge were used as reported previously [16]. Rats were actively sensitized with subcutaneous injection of 10 mg ovalbumin (OVA, Sigma, USA.) together with 200 mg aluminum hydroxide (Sigma, USA) in 1 ml 0.9% NaCl solution, and 1 ml inactivated bordetella pertussis vaccine (6 × 10^9^ heat-killed baciUin, Chengdu Institute of Biological Product, China) was administered intraperitoneally on the first day (Day 1). The rats were sensitized again on the eighth day (Day 8). Then the next six weeks the rats were exposed to aerosolized 2% OVA in 0.9%NaCl, which was generated by an ultrasonic nebulizer with 3 ml/min output, for 30 minutes and repeated three times a week. The total experimental time was eight weeks. After challenged, the rats showed symptoms of asthmatic attack such as agitation, bucking, tachypnea, cyanosis, and so on. In control group, the rats were sensitized and challenged with the same volume of 0.9% NaCl instead of OVA. In SMI treatment group, the rats were not only sensitized and challenged by OVA to make the chronic asthmatic model, but also injected intraperitoneally with 2 mL SMI every day. The effective dose of SMI was determined by our previous studies [15,17]. The rats were sacrificed within 18–24 hours after the final challenge in each group.

### Pathomorphological image analysis of airway remodeling

Lung tissue sections stained with Hematoxylin and Eosin (HE) in each group were selected and analyzed with 422BLK8079 High-resolution Medical Colorful Image Analyzing System (Yiming Technology Development Co., Ltd., Guangzhou, China). The airway internal perimeter (Pi), wall area (WA), the area of bronchial smooth muscle (S) and the number of bronchial smooth muscle nucleus (N) were measured. Then the above measured values were standardized by Pi and WA/Pi, S/Pi, N/Pi represented respectively.

### ASMC isolation, culture and identification

ASMC were cultured using the reported method with some improvement [18]. Male rats were deeply anesthetized with pentobarbital sodium (5 ml/kg ip) and euthanized by exsanguination according to the protocol approved by the Institutional Animal Care and Use Committees of Zhengzhou University, China. Large bronchi which dissected from the surrounding parenchyma were washed three times in precooled modified Krebs-Henseleit solution [(in mM): 118 NaCl, 4.7 KCl, 1.2 NaH_2_PO_4_, 25.5 NaHCO_3_, pH value adjusted to 7.4 with 1 mM NaOH], containing antibiotics (penicillin G 100 U/ml and streptomycin 100 U/ml). Airways without cartilages were selected and pure airway smooth muscle bundles were cut free from surrounding tissues with the aid of a dissecting microscope. Then the airway was cut open and the endothelium was disrupted by gently stripping the luminal surface with a blade. The minced tissue (1 mm^3^ fragments) were seeded into sterilized 25 ml culture flasks and 2-3 ml DMEM (Gibco, USA) containing 20% (v/v) fetal bovine serum (FBS, Gibco, USA) and 100 U/ml penicillin G and streptomycin was added. The flasks were placed in a 5% CO_2_ humidified incubator at 37°C. The culture medium was replaced regularly (2-3 days) until cell confluence occurred (usually 8-10 days). Then the cells were digested with 0.25% trypsin (containing 0.02% EDTA, Gibco, USA) and subcultured in DMEM with 10% FBS. Immunofluorescence staining for α-smooth muscle actin (α-actin) was used to identify the smooth muscle cells. Cells were used for experiment between passages 3 and 5.

### RT-PCR to detect the mRNA expression of TRPV1 channel and PCNA

RNA was isolated and purified using Trizol regent (Gibco, USA) according to the manufacturer’s instructions. cDNA was synthesized from mRNA with the aid of an omniscript reverse transcriptase (Qiagen, CA) using 100 ng of RNA per reaction. PCR amplification (40 cycles of 95°C for 30 s, 55°C for 30 s, 68°C for 1 min; 72°C for 5 min) was performed with 5 μl of cDNA using the specific TRPV1 primer (Qiagen QT00180782), PCNA primer (Qiagen QT00178647) and β-actin (Qiagen QT00193473), yielding a product of 104 bp, 93 bp and 145 bp respectively. The amplified PCR products were confirmed by electrophoresis on 1.5% agarose gel containing ethidium bromide. The straps of amplified products were analyzed with MUVB-20 gel analysis system (Ultralum Corporation, USA) and the absorbance value (value of A) of each strap was measured. The relative values of A of TRPV1 and PCNA were normalized to β-actin.

### Immunofluorescence staining to detect the protein expression of α-actin and TRPV1

Immunocytochemistry was used to study the expression of α-actin and TRPV1 proteins in primary rat ASMC. ASMC were fixed with 4% formaldehyde for 10 minutes, permeabilized with methanol (−80°C), blocked with 2% bovine serum albumin (in phosphate buffered saline), and incubated with a primary sheep monoclonal antibody specific to α-actin and rabbit monoclonal antibody specific to TRPV1(1:100, Santa Cruz Biotechnology Inc., USA) overnight at 4°C. Cells were washed and incubated with a 1:500 fluorescent anti-sheep secondary antibody conjugated with a FITC fluorophore (green fluorescence, Santa Cruz Biotechnology Inc., USA) for α-actin detection and anti-rabbit conjugated with Texas (red fluorescence, Santa Cruz Biotechnology Inc., USA) for TRPV1 detection for two hours at room temperature in the dark. Cell nuclei were counterstained with 4′,6-diamidino-2-phenylindole (DAPI, blue fluorescence, Santa Cruz Biotechnology Inc., USA). The fluorescence images for immunocytochemistry were obtained using a laser-scanning confocal microscope (Olympus). The excitation of FITC was by illumination at 488 nm, 561 nm for Texas and 405 nm for DAPI. For the measurement of TRPV1 channel expression, the mean fluorescence intensity was quantified using the Olympus micro-image 4.0 analyzing software (Japan). The mean fluorescence intensity was normalized to the control group.

### Determination of the intracellular Ca^2+^ concentration ( [Ca^2+^]i )

The [Ca^2+^]i was measured using the intracellular calcium indicator Fluo-3/AM (Invitrogen, USA). The ASMC were loaded with 5 μM Fluo-3/AM for 30 minutes at 37°C in the dark. After they were washed with physiological HEPES buffered solution (in mM): 146 NaCl, 4.7 KCl, 2.5 CaCl_2_, 0.6 MgSO_4_, 0.15 NaHPO_4_, 0.1 ascorbic acid, 8 glucose and 10 HEPES; (pH 7.4), the cells were viewed using a confocal microscope (400× oil immersion objective, Olympus, Japan). Fluo-3/AM fluorescence was excited at 488 nm and emitted at 510/530 nm. [Ca^2+^]i was calculated using the formula: [Ca^2 +^]i = Kd[F ‒ Fmin]/[Fmax ‒ F], where the Kd for Fluo-3/AM is 400nM, F is the actual fluorescence intensity of the cell, Fmin is the fluorescence intensity when the cell was treated with 3 mM EGTA (Sigma, USA), Fmax is the fluorescence intensity when the cell was incubated with HEPES buffered solution containing 10 mM CaCl_2._

### Detection of PCNA by immunocytochemistry

Streptavidin-perosidase (SP) method was used for PCNA immunohistochemical staining. Operate according to SP kit instructions. Diluted concentration of mouse monoclonal antibody specific to PCNA (Santa Cruz Biotechnology Inc., USA) was 1:100. Colored with diaminobenzidine (DAB, Gibco, USA). The positive staining of PCNA was brown color, mainly located in the nucleus. Randomly select five views of each cell coverslip, measure and analyze the optical density (value of OD), then calculate the average value of OD of each group. Set the relative value of OD of the control group as 1.0, and the relative value of OD of the other two groups was normalized to the control group.

### 3-(4,5-Dimethylthiazol-2-yl)-2,5-diphenyltetrazolium bromide (MTT) assay to detect the cell proliferation

Rat ASMC were digested with 0.25% trypsin-EDTA and then seeded into 96-well culture plate with the density of 1 × 10^4^/ml. After two days, 12 mM MTT stock solution (Gibco, USA) was added to each well. Incubate at 37°C for 4 hours. Remove the entire medium from the wells, add 150 μL of dimethyl sulfoxide (DMSO, Sigma, USA) to dissolve the colored formazan crystals, and mix thoroughly with the oscillator for 10 minutes. Then read absorbance at 490 nm wavelength (value of A_490_) with an enzyme-linked immunosorbent assay reader (Bio-tek ELX800, USA). The value of A_490_ is proportional to the number of living cells. Each group had six wells.

### Statistical analysis

All data were expressed as mean ± SD $\left(\bar{x}\pm s\right)$. SPSS 16.0 software was used for statistical analysis. Differences among several groups were determined with the One-way ANOVA. Differences were considered significant with P < 0.05. Sigma Plot v11.0 was used to compile the figures.

## Results

### Pathomorphological image of airway remodeling

**Figure 1:**
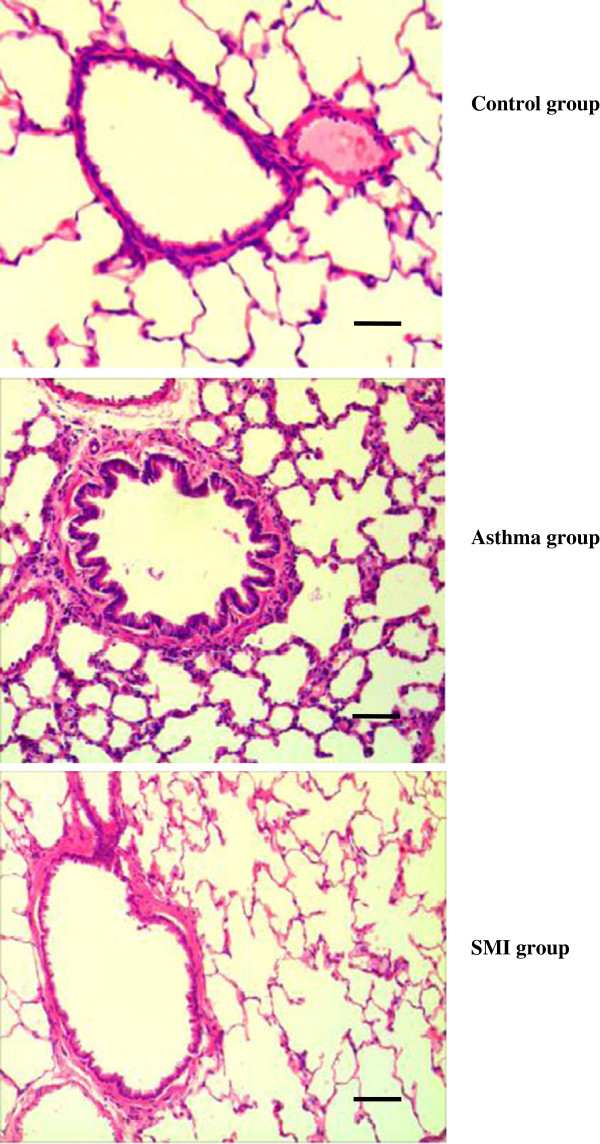
**The bronchial smooth muscle of each group (HE staining).** The thickness of ASM in chronic asthma rats was significantly increased. After SMI treatment, the over-proliferation of ASM was significantly inhibited. Bar = 20 μm.

In chronic asthmatic rats, the mucosal fold membranes of bronchiole were increased and broken. A large number of eosinophils infiltrated into the bronchial submucosa and the thickness of airway wall and bronchial smooth muscle were significantly increased. That meant airway remodeling was formed in the chronic asthmatic model. But after SMI treatment, airway remodeling was significantly reduced (Figure 1). WA/Pi, S/Pi, N/Pi of each group were shown in Table 1.

**Table 1:** **Comparison of WA/Pi, S/Pi, N/Pi of each group** ($\left(\bar{x}\pm \;s,\;n=10\right)$

**Group**	**WA/Pi (μm**^**2**^**/μm)**	**S/Pi (μm**^**2**^**/μm)**	**N/Pi (n/μm)**
Control group	7.02 ± 0.31	2.57 ± 0.24	0.0181 ± 0.0042
Chronic asthma group	11.82 ± 1.37**	3.71 ± 0.47**	0.0388 ± 0.0162*
SMI group	7.74 ± 0.29^# #^	2.68 ± 0.34^#^	0.0184 ± 0.0056^#^
F	63.245	17.923	8.136
P	<0.001	0.005	0.029

*P < 0.05, **P < 0.01 vs. the control group.

^#^P < 0.05, ^# #^P < 0.01 vs. the chronic asthma group.

### Characteristics and identification of rat ASMC in primary culture

**Figure 2:**
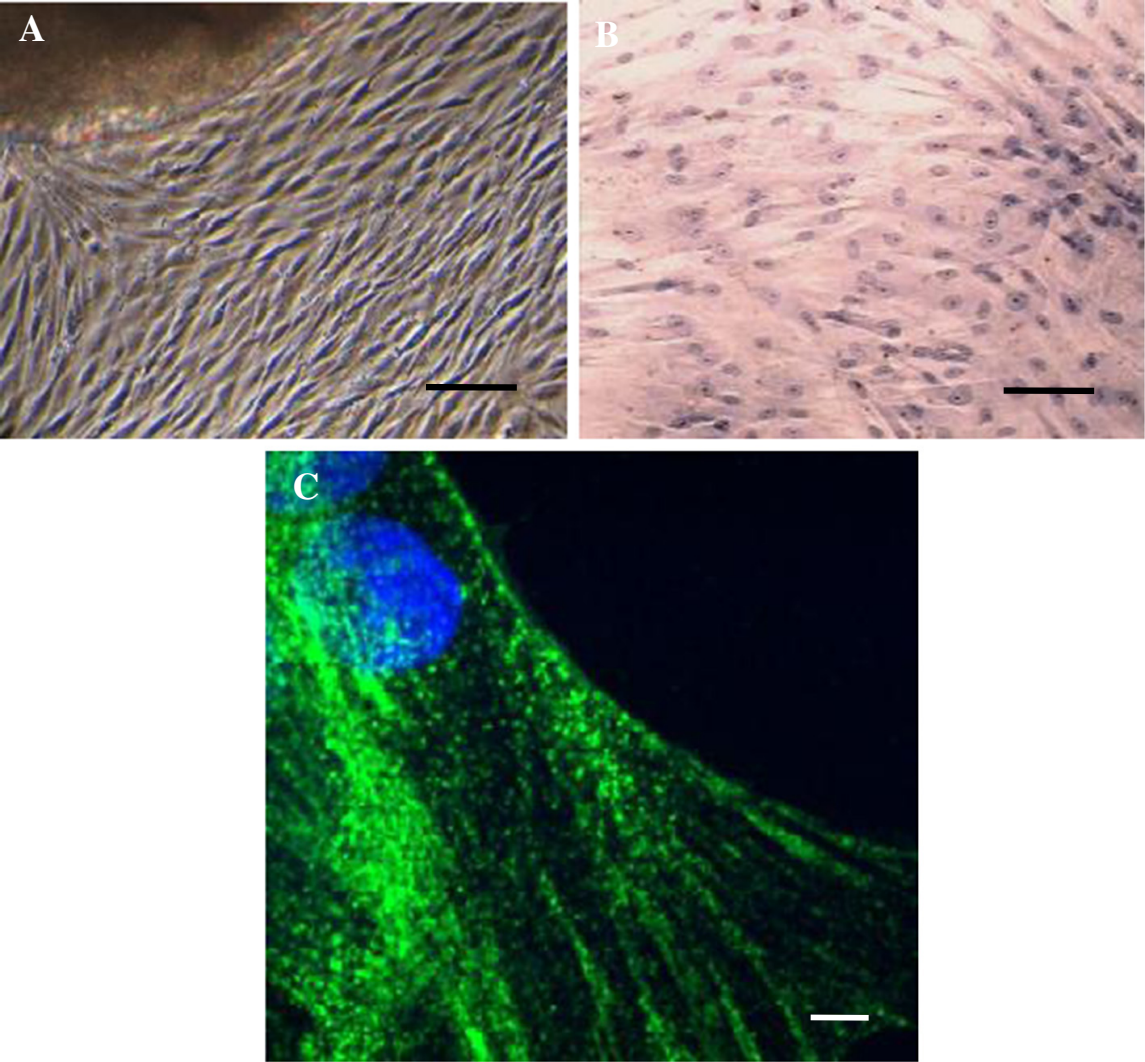
**Primary rat ASMC. A**: Cells growing around the tissues with spindle-shaped, cell body and central oval nuclei in 2–3 days, then cells confluence occurring in 8-10 days (phase-contrast microscopy). Bar = 100 μm. **B**: Typical “hill and valley” appearance in ASMC (HE staining). Bar = 50 μm. **C**: Positive α-actin fluorescent immunostaining with green parallel fiber in cytoplasm. Bar = 10 μm.

In phase-contrast imaging, cells cultured for 2–3 days from airway appeared to be spindle-shaped and had central oval nuclei with prominent multiple nucleolus. In 8–10 days, the cells began to merge and displayed the typical “hill and valley” appearance of smooth muscle cells (Figure 2A and B). α-actin was detected by immunofluorescence staining and appeared positively as green parallel fiber in cytoplasm (Figure 2C).

### Expression of TRPV1 in ASMC of each group

**Figure 3:**
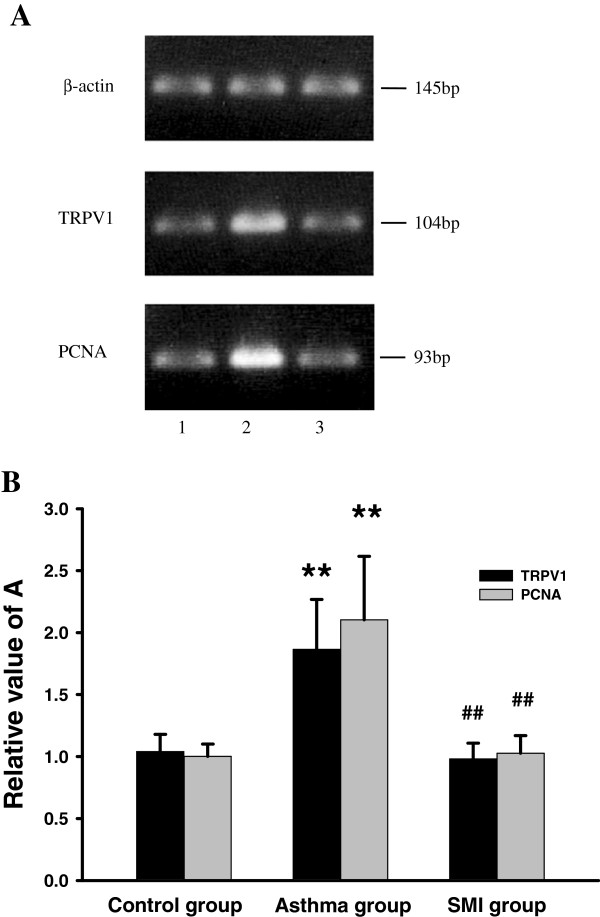
**The mRNA expression of TRPV1 and PCNA in ASMC of each group. (A)** RT-PCR of TRPV1, PCNA and β-actin in rat ASMC of each group. 1. Control group; 2. Asthma group; 3. SMI group. **(B)** Summary of mRNA expression of TRPV1 and PCNA in ASMC of each group (n = 8). The relative values of A of TRPV1 and PCNA were normalized to β-actin. **P < 0.01 vs. the control group. ^# #^P < 0.01 vs. the asthma group.

The mRNA expression of TRPV1 in asthmatic rat ASMC significantly increased compared with the control group [the value A from (1.04 ± 0.14) to ÿ1.87 ± 0.41, P < 0.01]. And after SMI treatment, the expression of TRPV1 in asthmatic rat ASMC (0.98 ± 0.13) was significantly decreased (P < 0.01) (Figure 3A,B).

**Figure 4:**
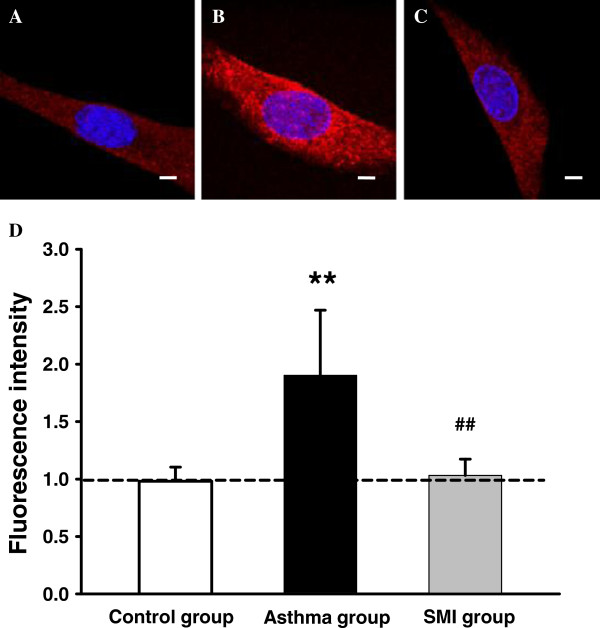
**Expression of TRPV1 protein in rat ASMC of each group.** Representative images of immunofluorescent labeling for TRPV1 in rat ASMC of control group **(A)**, chronic asthma group **(B)** and SMI group **(C)**. The positive staining of TRPV1 was red fluorescence in membrane and cytoplasm.Nuclei were dyed with blue. Bar = 10 μm. **D**. Summarized data of TRPV1 protein expression in ASMC of each group. The mean fluorescence intensity of control group was set to 1.0. The mean fluorescence intensity of the other two groups was normalized to control group. **P < 0.01 vs. the control group. ^# #^P < 0.01 vs. the asthma group.

TRPV1 protein was detected by immunofluorescence labeling. The red fluorescence signals were observed with a fluorescent confocal microscope (Figure 4A,B and C). As shown in Figure 4D, the protein expression of TRPV1 in asthmatic rat ASMC was significantly increased [the value A from (0.98 ± 0.12) to ÿ1.90 ± 0.57), P < 0.01]. In the SMI treatment group, the protein expression of TRPV1 channel (1.03 ± 0.14) was significantly decreased in ASMC (P < 0.01).

The result showed that SMI significantly inhibited the expression of TRPV1 channel.

### [Ca^2+^]_i_ in ASMC

**Figure 5:**
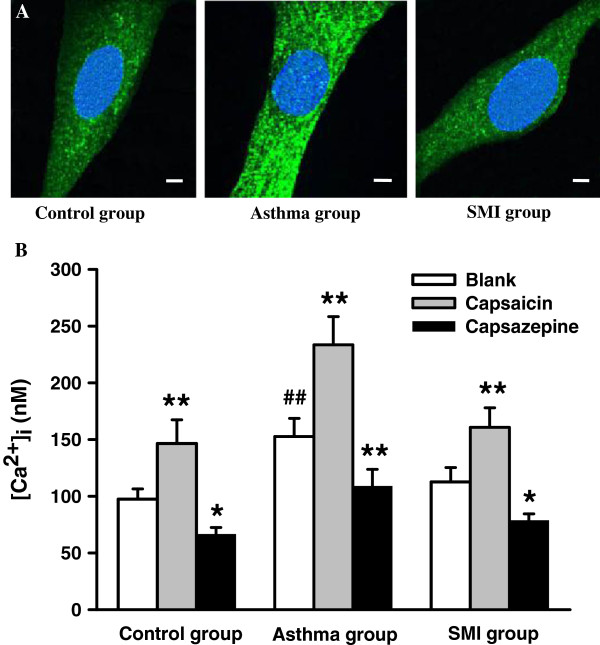
**Intracellular Ca**^**2+ **^**concentration ([Ca**^**2+**^**]**_**i**_**) in rat ASMC of each group.** Representative images of intracellular Ca^2+^ fluorescence in ASMC of each group. ASMC were incubated with Fluo-3/AM, so the green fluorescences were observed in the cells. The fluorescence intensity in asthmatic ASMC was increased compared with the control group, and SMI could diminish the fluorescence intensity. Bar =10 μm **(A)**. Summarized data showed that TRPV1 selective agonist capsaicin increased [Ca^2+^]_i_ and inhibitor capsazepine decreased [Ca^2+^]_i._ [Ca^2+^]_i_ of asthmatic ASMC were significantly increased and [Ca^2+^]i was reduced down to the normal level in SMI group (n = 20). *P < 0.05, **P < 0.01 vs. the blank group . ^# #^P < 0.01 vs. the blank control group **(B)**.

ASMC were incubated with Fluo-3/AM, so the green fluorescences were observed in the cells. The fluorescence intensity in asthmatic ASMC was increased compared with the control group, and SMI could diminish the fluorescence intensity (Figure 5A). TRPV1 selective agonist capsaicin increased [Ca^2+^]_i_ and inhibitor capsazepine decreased [Ca^2+^]_i._ So TRPV1 channel was involved in regulating [Ca^2+^]_i_ of ASMC. [Ca^2+^]_i_ of asthmatic ASMC were significantly increased and [Ca^2+^]_i_ was reduced down to the normal level in SMI group (n = 20). Therefore SMI reduced the [Ca^2+^]_i_ in asthmatic rat ASMC (Figure 5B).

### SMI inhibited the proliferation of asthmatic rat ASMC

Immunostaining, ×400

### Expression of PCNA in ASMC of each group

**Figure 6:**
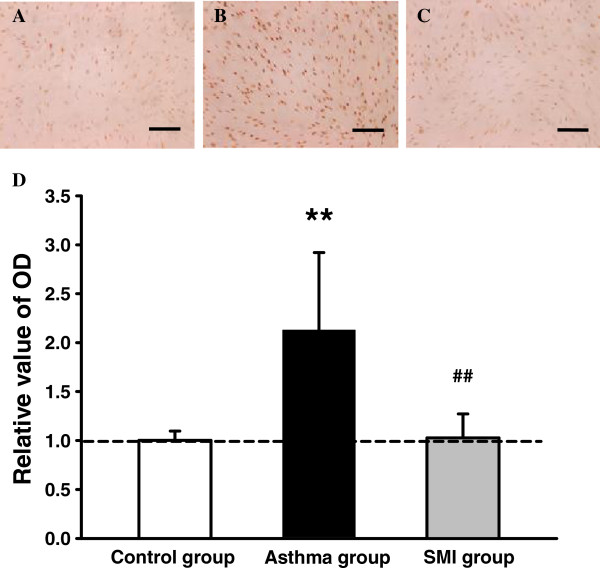
**Expression of PCNA in rat ASMC of each group.** Representative images of immunocytochemistry for PCNA in rat ASMCs of control group **(A)**, chronic asthma group **(B)** and SMI group **(C)**. The positive staining of PCNA was brown color, mainly located in the nucleus. Bar = 100 μm. **D**. Summarized data of PCNA protein expression of ASMC of each group. Set the relative value of OD of the control group as 1.0, and the relative value of OD of other two groups was normalized to the control group. **P < 0.01 vs. the control group. ^# #^P < 0.01 vs. the asthma group.

As shown in Figure 3 and Figure 6, the mRNA and protein expression of PCNA in asthmatic rat ASMC were significantly increased compared with the control group. Whereas SMI could inhibit the increase of PCNA expression induced by asthma (P < 0.01).

### The results of MTT assay

The value of A_490_ of each group was shown in Table 2. The value A_490_ of asthma group was significantly increased. After SMI treatment, the value A_490_ was decreased. That meant that SMI could inhibit the ASMC over-proliferation caused by asthma ( P <0.05).

**Table 2:** **MTT absorbance of each group**$\left(\bar{x}\pm \;s,\;n=10\right)$

**Group**	**Well**	**Value of A**_**490**_
Control group	6	0.296 ± 0.024
Chronic asthma group	6	0.535 ± 0.087*
SMI group	6	0.312 ± 0.036^#^
F		33.912
P		0.001

*P < 0.05 vs. the control group.

^#^P < 0.05 vs. the chronic asthma group.

## Discussion

Asthma is a common chronic inflammatory respiratory disease over the globe, linking to 250, 000 cases of deaths each year [19]. It is characterized by airway inflammation, airway wall remodeling and airway hyper-responsiveness (AHR). Moreover, airway wall remodeling can lead to airway inflammation and AHR [20]. As a characteristic of pathological change in asthma; airway remodeling was also the pathological basis of chronic, persistent, severe, steroid-resistant asthma, and irreversible airway obstruction [21-24].

Airway remodeling refers to structural changes such as bronchial fibrosis, increase in basal membrane thickness, smooth muscle hyperplasia and hypertrophy and so on [25]. In particular, smooth muscle remodeling has been associated with a decrease in lung function leading to a more severe asthma phenotype [21,26]. Therefore, studying the mechanism of ASMC proliferation is essential in asthma research.

The transient receptor potential ion channel protein (TRP) is a class of transient non-selective receptor proteins for Ca^2+^ cations present in cell membranes or intracellular organelle membranes. The TPR family includes seven sub-families: TRPC, TRPV, TRPM, TRPML, TRPP, TRPA and TRPN channels. In particular, TRPV is a major sub-family with at least six known members in mammals, namely TRPV1-6 [27,28]. Among them, TRPV1 channel is the first cloned [29], more studied and more popular channel. Therefore in this study, we focused on the TRPV1 channel in ASMC.

The TRPV1 channel is presented in various parts of the mammalian respiratory system, including the trachea sensory nerves, ASMC, epithelial cells, pulmonary vascular endothelial cells, pulmonary artery smooth muscle cells, sub-mucosal glands, and inflammatory cells [30]. In recent years, studies showed that TRPV1 channel played a crucial role in the cough reflex. When the TRPV1 channel was activated, which induced Ca^2+^ influx into the nerve cells, causing membrane depolarization to reach the threshold potential, which in turns led to bronchoconstriction, mucus secretion and the cough reflex [31]. In addition, the Ca^2+^ influx caused by TRPV1 channel activation could trigger the release of tachykinin (TK) and calcitonin gene-related peptide (CGRP) in peripheral and central nervous system, resulting in brochoconstriction, protein secretion, tracheal mucosal edema and inflammatory cell chemokines [32]. Cantero’s report [10] showed that TRPV1 channel was highly relevant to asthma because the level and activity of the genetic determinants of TRPV1 channel affected the occurrence and pathophysiological processes of asthma. In another study, it was found that chronic hypoxia significantly increased the mRNA and protein expression of TRPV1 channel in human pulmonary artery smooth muscle cells, leading to an increase in cytosolic Ca^2+^ concentration and thus cell over-proliferation [33]. However, the role of TRPV1 channel in ASMC proliferation of asthma has not been reported.

For that reason, we have studied the TRPV1 channel in rat ASMC and found that the TRPV1 channel was one of the important channels to regulate calcium influx in rat ASMC. The TRPV1 expression was observed to increase in the asthmatic rat ASMC, leading to an increase in the intracellular calcium concentration, which in turn resulted in an increase of the expression of PCNA. PCNA was chosen as a marker for proliferation. The PCNA expression could reflect the state of cell proliferation. When the PCNA expression was increased, cell proliferation was promoted. Conversely, cell proliferation would be inhibited when the PCNA expression decreased [34]. So, as a consequence, the ASMC showed excessive proliferation and thus asthma-induced airway remodeling occurred.

As the understanding of SMI deepens, more and more attention has been given to the clinical applications of SMI in the treatment of respiratory system diseases. SMI has been widely used in the treatment of chronic obstructive pulmonary disease (COPD), asthma, pulmonary heart disease [12], lung cancer patients [35] and so on.

This study found that SMI could down-regulate the expression of TRPV1 channel from an abnormally high level to almost normal level in asthmatic rat ASMC. At the same time the level of intracellular calcium and the expression of PCNA also returned to normal in these ASMC. Therefore, the over-proliferation of ASMC caused by asthma was inhibited by SMI.

## Conclusions

In summary, these findings suggest that SMI may effectively inhibit the progression of asthma-induced ASMC remodeling. This anti-remodeling effects of SMI were probably mediated partially by downregulation of TRPV1 channel. Although clinical applications require further investigation, this study has provided the scientific experimental basis for SMI to prevent and treat the airway remodeling in asthma.

## Competing interests

The authors declare that they have no competing interests.

## Authors’ contributions

LZ, LM and JW participated in the design of the study data analyses and manuscript preparation. XZ, HK and YG conducted the assays and analyses. All authors read and approved the final manuscript.

## Pre-publication history

The pre-publication history for this paper can be accessed here:

http://www.biomedcentral.com/1472-6882/13/221/prepub
